# Augmented Repair of Degenerative Tears of Tendo Achilles Using Peroneus Brevis Tendon: Early Results

**DOI:** 10.5704/MOJ.1303.011

**Published:** 2013-03

**Authors:** Akhil A Tawari, Anoop C Dhamangaonkar, Arvind B Goregaonkar

**Affiliations:** Department of Orthopaedics, Lokmanya Tilak Municipal Medical College and General Hospital, Mumbai, India; Department of Orthopaedics, Lokmanya Tilak Municipal Medical College and General Hospital, Mumbai, India; Department of Orthopaedics, Lokmanya Tilak Municipal Medical College and General Hospital, Mumbai, India

## Abstract

Reconstruction of degenerated ruptures of the tendoachilles is a challenge. Ruptured tendons and the remaining tendon ends are abnormal. A number of methods have been described in literature reconstruct the tendoachilles, but with variable results^1^. We used peroneus brevis tendon in 20 patients to augment the repair of degenerated tendoachilles tears by creating a dynamic loop as described by Teuffer et al^2^. All patients were followed up for atleast 18 months. At the last postoperative visit, 18 out of 20 patients were able to do a toe raise. Eighty-five per cent of patients had excellent or good results and 15% had fair or poor results using modified Rupp scoring. Advantages offered by this procedure are the use of a single incision and mini incision and use of a dispensable tendon such as the peroneus brevis without entirely depending on the damaged tendon for healing.

**Key Words:**

Degenerative tear of tendoachilles, augmented repair, peroneus brevis tendon

## Introduction

Degenerative ruptures of tendoachilles typically occur after the age of 30 years. An inciting event may be related to atrophy of the tendon as commonly occurs in weekend athletes. The injury mechanism usually involves eccentric loading on a dorsiflexed ankle with the knee extended^4^,^5^. The Achilles tendon has no true synovial sheath, unlike the flexor tendons of the hand; rather, it is covered only by a paratenon and exogenous healing (from synovial fluid) is not expected to occur. Side effects of gout, hyperparathyroidism, steroids and flouroquinolones may contribute to tendon rupture^6^. In the past, we initially treated this injury with end suturing and a plaster cast, but this was associated with high rates of reruptures and weakened push off. Hence, there is rationale to perform reconstruction using an expendable yet healthy tendon such as the peroneus brevis . Here, we present a study of twenty patients treated with this technique.

## Materials and Methods

Twenty two patients with a degenerative tendo achilles tear were repaired using peroneus brevis tendon between may 2006 and January 2011 . Two patients were lost to follow up. All the patients presented acutely or within a few days due to inability to walk normally post-injury. Clinical presentation was typical with pain and a snapping sensation behind the ankle following a sudden jerk while engaging in sports or similar activity. The patients complained of difficulty in walking and inability to run. Clinical examination revealed local site tenderness, inability to actively plantarflex the ankle (passive plantarflexion was possible) and positive Thompsons’ test^7^. Ankle radiographs were obtained to rule out calcaneal fractures; patients with such fractures were excluded from the study. All patients underwent operative treatment after giving written informed consent.

With the patient in prone position, a posterolateral longitudinal incision was made along the tendoachilles also exposing the calcaneal tuberosity.The sural nerve was identified and retracted proximally in the wound. Incision was made through the tendoachilles sheath to expose the ruptured ends (Figure 1a). Scar tissue was resected and the tendon dissected proximally to free it if needed (Figure 1b). The peroneus brevis was then detached from its insertion on the fifth metatarsal following a mini incision and brought through to the first wound (Figure 1c). Ruptured tendon ends were approximated using the modified Krackows’ technique with No. 2 ethibond suture (Figure 1d). We then drilled a hole large enough for the peroneus brevis through the transverse diameter of the calcaneal tuberosity. The peroneus brevis was passed through this hole and then back proximally beside the site of rupture for reinforcement; finally, it was sutured to itself to produce a dynamic loop similar to modified Teuffer technique (Figure 1e). Patients were put in a plaster cast with the ankle in 10-15° plantarflexion and the knee in 15 degree of flexion for 4 weeks. This was followed by a below knee cast with the ankle in neutral position for another 4 weeks. Weight bearing was started 6 weeks post-operatively and cast was discontinued 8 weeks post operatively. A progressive strengthening rehabilitation programme followed.

## Results

Of the 20 patients , 12 were 12 female and 8 male, and average age was 41 years (range, 38-51 y). Three patients were on long term steroids for respiratory complaints, one had gout , and the remaining patients had no significant medical or surgical history. All patients were followed up for at least 18 months. (range, 19-48 months) (Table I).

All patients were asked return for an evaluation by one of the authors who was not involved in the surgical management of any of the cases, and were examined using objective and subjective criteria. Objectively, ankle range of motion, ability to perform a toe raise, and neurological status of the foot were examined. Subjective criteria included the Rupp score, as modified by Kerkhoffs et al. (Table IV). In addition to information gathered in the follow-up interview, nformation was also gathered from the patients’ medical record. Results were rated as excellent (>30 points), good (15-30 points), fair (5-15 points) and poor (<5 points) (Table II).

Average dorsiflexion was 18° (compared to 24°on the noninjured side) and average plantarflexion was 26° ( compared to 35°on the non-injured side ). Resuts of testing the patient’s ability to toe raise for 60 seconds, 13 patients were able to sustain, while 5 patients were able to raise the toe but could not sustain it. Two patients could not do raise the toe at all. Three patients complained of sensory hypoesthesia at 18 months follow-up. For Rupp scoring , 85 % patients had excellent or good results and 15% had fair or poor results.

One patient suffered a re-rupture, but refused further surgery and was managed using ankle foot orthosis. Another patient had a superficial postoperative infection, which was managed with debridement followed by wound closure using free flap and needing plastic surgery intervention. Two of patients developed hypertrophic scarring and have problems with footwear. (See Table III, complications)

## Discussion

Treatment of a degenerative tendoachilles tear is a tricky proposition. Results of Achilles tendon repair have been variable. As noted by Lagergren and Lindholm^8^, the tendoachilles region 2 to 6 cm above the calcaneal insertion has the poorest blood supply. Carr and Norris^9^ demonstrated that the midsection of the tendon is most prone to rupture, as this is the area of the tendon in which there is a reduced percentage and number of blood vessels. In addition, the tendo achilles is devoid of a true synovial sheath and has only a paratenon which is more prone to inflammation. Histological examination of ruptured tendon ends confirmed these findings^4^. In the present study, all but one study participant had prodromal symptoms of tendonitis in the form of pain, and reported either acutely or within a few days of onset of inability to walk properly.

There are many treatment options for Achilles tendon rupture and many have long been a matter of controversy, including closed methods^10^,^11^, open surgical repair, percutaneous sutures^12^, v-y lengthening of the gastrocnemius^13^, augmented repair with central gastrosoleus aponeurosis^1^, and reconstruction using flexor hallucis longus^14^,^15^. We performed reconstruction using peroneus brevis based on the premise that the torn ends of the tendons are already unhealthy^4^. Further, the healing capacity of the injured tendon is further limited due to hypovascularity resulting in decreased tissue regeneration with a high probability of re-reupture. The use of of peroneus brevis serves two advantages: 1) it incorporates a healthy tendon with more reliable healing potential; 2) it is an expendable tendon and there is little disability in its absence. Overall, our results were satisfactory withn 85% good or excellent results as per modified Rupp criteria. Similarly, Teuffer^2^ et al. reported that this is a dynamic loop repair technique which is biomechanically more sound than static repair.

Nevertheless achilles tendon reconstruction using peroneus brevis has certain diadvantages. For instance, this more extensive approach requires specialized surgical expertise. Infection, though rare is a pssibility. Superficial infection and skin loss occurred in one patient in the present study and was managed with thorough debridement and free flap. Altered wound healing in the form of hypertrophic scarring can result into difficulty in shoe wearing.

Similar augmented techniques are reported in the literature. For instance, Demirel et al.^1^ noted that primary repair of acute tendo achilles rupture augmented with thegastrosoleus turn down flip technique in combination with immediate weightbearing ambulation results in good outcomes overall, but is associated with similar complication rates noted above.

There are a number of shortcomings of our study. Firstly, the sample size of 20 patients is too low. Also, no one in the present study was a professional athlete, members of a subpopulation who would likely have higher expectations for such a procedure.

**Table I:**
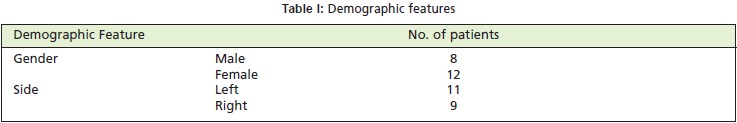
: Demographic features

**Table II:**
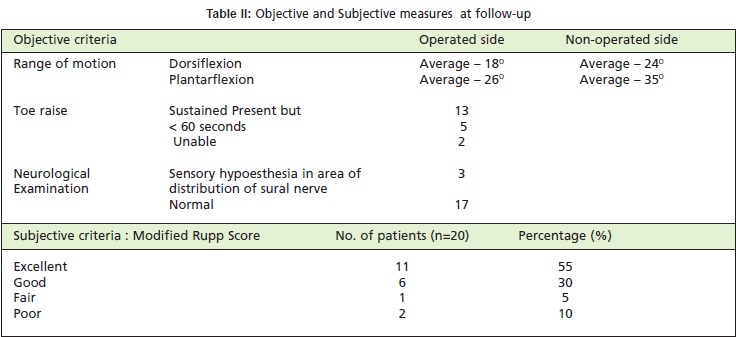
: Objective and Subjective measures at follow-up

**Table III:**
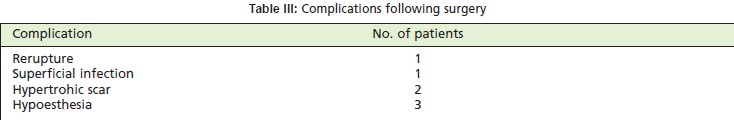
: Complications following surgery

**Table IV:**
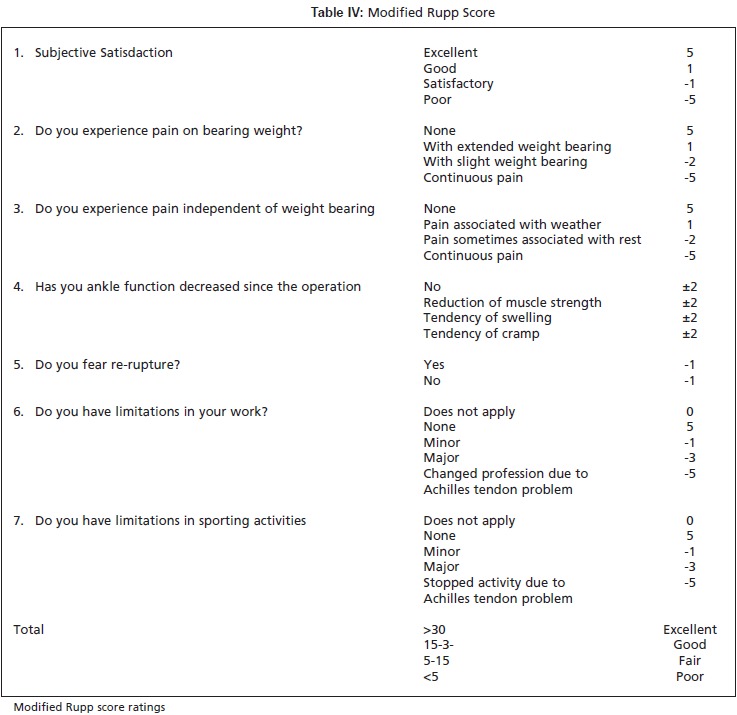
: Modified Rupp Score

**Figure 1a:**
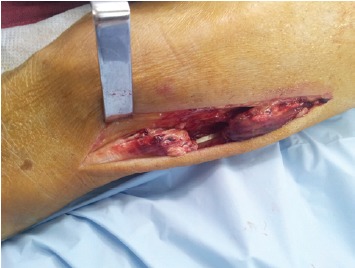
: Photograph showing incised tendo achilles sheath and torn tendon.

**Figure 1b:**
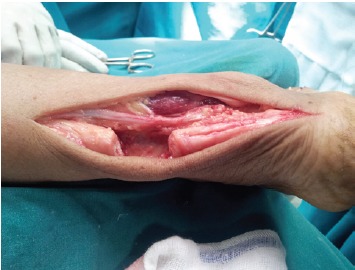
: Photograph showing tendon dissected proximally with freshened torn ends and freed sural nerve.

**Figure 1c:**
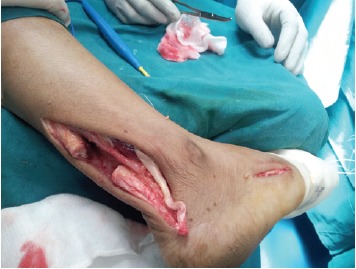
: Photograph showing freshened torn ends of tendo achilles and peroneus brevis harvested from insertion via a mini incision and brought through to the primary wound.

**Figure 1d:**
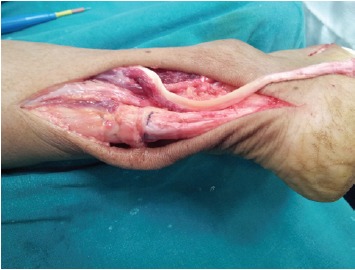
: Photograph showing repair of tendo achilles using Krackow’s technique.

**Figure 1e:**
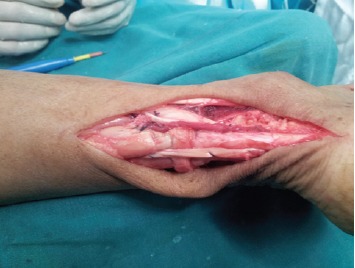
: Augmentation of repair using peroneus brevis and modified Teuffer technique. A hole large enough for the peroneus brevis to pass through the transverse diameter of the calcaneal tuberosity. The peroneus brevis was passed through this hole and then back proximally beside the site of rupture for reinforcement; finally, it was sutured to itself to produce a dynamic loop similar to modified Teuffer technique.

## Conclusion

Results of reconstruction of Achilles tendon ruptures using peroneus brevis tendon show a strong and stable repair that allows early weightbearing ambulation with favorable clinical results in most patients. Disadvantages of the procedure should be shared in detail with patients when obtaining informed consent. Care must be taken to prevent wound problems and deep infection that can necessitate more extensive dissection. Further studies that include professional athletes should be performed to confirm efficacy of this augmented technique.
